# High-impact and transformative science (HITS) metrics: Definition, exemplification, and comparison

**DOI:** 10.1371/journal.pone.0200597

**Published:** 2018-07-19

**Authors:** Joseph Staudt, Huifeng Yu, Robert P. Light, Gerald Marschke, Katy Börner, Bruce A. Weinberg

**Affiliations:** 1 Ohio State University, Columbus, Ohio, United States of America; 2 University at Albany, State University of New York, Albany, New York, United States of America; 3 CNS, School of Informatics, Computing, and Engineering, Indiana University, Bloomington, Indiana, United States of America; 4 National Bureau of Economic Research, Cambridge, Massachusetts, United States of America; 5 Indiana Network Science Institute, Indiana University, Bloomington, Indiana, United States of America; CPERI, GREECE

## Abstract

Countries, research institutions, and scholars are interested in identifying and promoting high-impact and transformative scientific research. This paper presents a novel set of text- and citation-based metrics that can be used to identify high-impact and transformative works. The 11 metrics can be grouped into seven types: Radical-Generative, Radical-Destructive, Risky, Multidisciplinary, Wide Impact, Growing Impact, and Impact (overall). The metrics are exemplified, validated, and compared using a set of 10,778,696 MEDLINE articles matched to the Science Citation Index Expanded^TM^. Articles are grouped into six 5-year periods (spanning 1983–2012) using publication year and into 6,159 fields constructed using comparable MeSH terms, with which each article is tagged. The analysis is conducted at the level of a field-period pair, of which 15,051 have articles and are used in this study. A factor analysis shows that transformativeness and impact are positively related (*ρ =* .*402*), but represent distinct phenomena. Looking at the subcomponents of transformativeness, there is no evidence that transformative work is adopted slowly or that the generation of important new concepts coincides with the obsolescence of existing concepts. We also find that the generation of important new concepts and highly cited work is more risky. Finally, supporting the validity of our metrics, we show that work that draws on a wider range of research fields is used more widely.

## 1. Introduction

Countries, research institutions, and scholars have prioritized high-impact and transformative scientific research. The National Science Board (NSB) argues that while research with the potential to transform science “is inherently less predictable in its course and eventual outcomes, it is, nonetheless, absolutely essential for our national advancement and for the advancement of science as a whole [1]”.

Recognizing the importance of transformative research, the National Institutes of Health (NIH) and National Science Foundation (NSF) both instituted initiatives to support transformative research. However, no standard metrics exist to identify transformative research. Such metrics are essential if we want to answer even such fundamental questions as: How frequent is transformative research? How important is transformative research for scientific progress? Does the prevalence of transformative research vary over time or across fields? To what extent are impact and transformativeness related? How do the demographics (in terms of gender, race, age, national origin) of fields, the structure of scientific networks, or the funding environment affect the production, diffusion, and reception of transformative research?

A National Science Board report from 2007 argues:

Science progresses in two fundamental and equally valuable ways: The vast majority of scientific understanding advances incrementally, with new projects building upon the results of previous studies or testing long-standing hypotheses and theories. This progress is evolutionary—it extends or shifts prevailing paradigms over time. The vast majority of research conducted in scientific laboratories around the world fuels this form of innovative scientific progress. Less frequently, scientific understanding advances dramatically, through the application of radically different approaches or interpretations that result in the creation of new paradigms or new scientific fields. This progress is revolutionary, for it transforms science by overthrowing entrenched paradigms and generating new ones. The research that comprises this latter form of scientific progress … [is] termed transformative research … [1].

We begin by grounding our work in established conceptualizations of transformative research from NIH, NSB, and NSF. These conceptualizations identify seven aspects of transformative work. Transformative work is seen to: (1) generate important new ideas (radical generative) and (2) make existing ideas obsolete or less salient (radical destructive), (3) be risky, (4) be multidisciplinary, (5) have a broad impact, (6) have an impact that builds over time, and (7) have a high impact. We then use rich characterizations of citations and text to develop eleven metrics that operationalize these seven aspects of transformative work (we develop multiple metrics for some aspects of transformativeness). We next use factor analysis to identify the combination of our eleven metrics that best characterizes the seven aspects of transformative work. Finally, we reduce the dimensionality of the metrics (other than impact) into a single measure of transformativeness. Our goal is to identify the scientific fields and periods of time in which high-impact work and/or transformative work was done, so our unit of analysis is “field-period pairs,” although many of these metrics can be computed for individual articles.

The behavior of our metrics of transformativeness largely correspond to existing conceptualizations but provide quantitative insights. Conventional citation measures of impact (aspect 7) are related to transformativeness, but our metrics show substantial independent variations in transformativeness (aspects 1–6) for a given level of impact (the partial correlation is .*402* across field-period pairs after eliminating all field and period effects). Thus, impact and transformativeness are empirically (as well as conceptually) distinct, each representing a distinctive, cohesive phenomenon. Looking at the subcomponents of transformativeness, we find that radical generative and radical destructive work (aspects 1 and 2) only moderately coincide, so that it is possible to generate large amounts of knowledge without obsolescing large amounts of existing knowledge. Radical generative work and works that are highly impactful (aspects 1 and 7) are both riskier (aspect 3). Strikingly, we find that transformative work has a shorter time to utilization (aspect 6, as measured by citations). Supporting the validity of our metrics, we show that work that draws on a wider range of research fields (aspect 4) is used across a wider range of fields. However, we find only a weak relationship between multidisciplinarity and impact (aspects 4 and 7).

It is worth noting that, while our data span a substantial time, 30 years, both the citation- and text-based metrics used to operationalize the seven aspects of transformativeness depend on scientists’ opinions of work during this period. Thus, some of what we identify as transformative may be fads that fail to become truly transformative. Mistaking a fad for transformative research becomes more likely as the end of our sample period approaches. It is also worth noting that the scientific enterprise is expanding during this period, which may make it possible for radical generative work to take place without the destructiveness that a more stagnant environment might involve.

Three areas of research that perform highly on transformativeness and (in most cases) also on impact were used to highlight the utility of the proposed metrics. Research on stem cells and epigenomics both rank highly on transformativeness and impact and, as discussed below, both are widely viewed as transformative. The Human Genome Project helped layhelp the foundation for the genomic revolution and advances in biotechnology. Strikingly, it ranks particularly highly on transformativeness (relative to impact).

When measuring scientific output and creativity, social scientists rarely use measures beyond publication counts, perhaps weighted by some journal ranking, and citation counts, which do not adequately distinguish work that is influential within a paradigm from work that is influential and also path-breaking and therefore do not allow separate analysis of impact and transformativeness in science. Recent work has sought to address deficiencies of standard citation methods (e.g., [2, 3]); has used a range of rich characterizations of citations to identify the most innovative work [4]; has identified novel research from unique combinations of citations [5]; and has used shifts in citation patterns to identify work that consolidates or destabilizes existing technologies [6]. An overview of approaches to identifying novelty and develop a unifying simulation approach can be found in [7].

Reviews of a wide range of scholarly metrics that are commonly used in citation and scholarly impact analysis but also in academic auditing can be found in [8,9]. While traditional metrics use a quantitative analysis of publications, authors, bibliographic references, and related concepts, novel metrics also consider text, acknowledgments, endorsements, downloads, recommendations, blog posts, and tweets. They argue that multi-dimensional metrics—also called mixed indicators—are most valuable as the performance of a person, institution, or country cannot be adequately measured by any single indicator. This is in line with [10], which compared 39 existing and proposed metrics of scholarly impact calculated on the basis of both citation and usage log data. They performed a principal component analysis of the rankings produced by these metrics to investigate how the different metrics relate to each other, and how accurately and completely they express scientific impact. They too conclude that the notion of scientific impact is a multi-dimensional construct and that multiple metrics are needed to cover impact. Recent work has developed and validated mixed indicators that help identify emerging research areas [11]. Other work has used the evolution of scientific collaboration networks to trace the evolution of fields [12]. The work presented in this paper is novel as it focuses on the development of metrics that support the identification of high-impact and transformative science (HITS).

## 2. Conceptualization of transformative work

**Fig 1:**
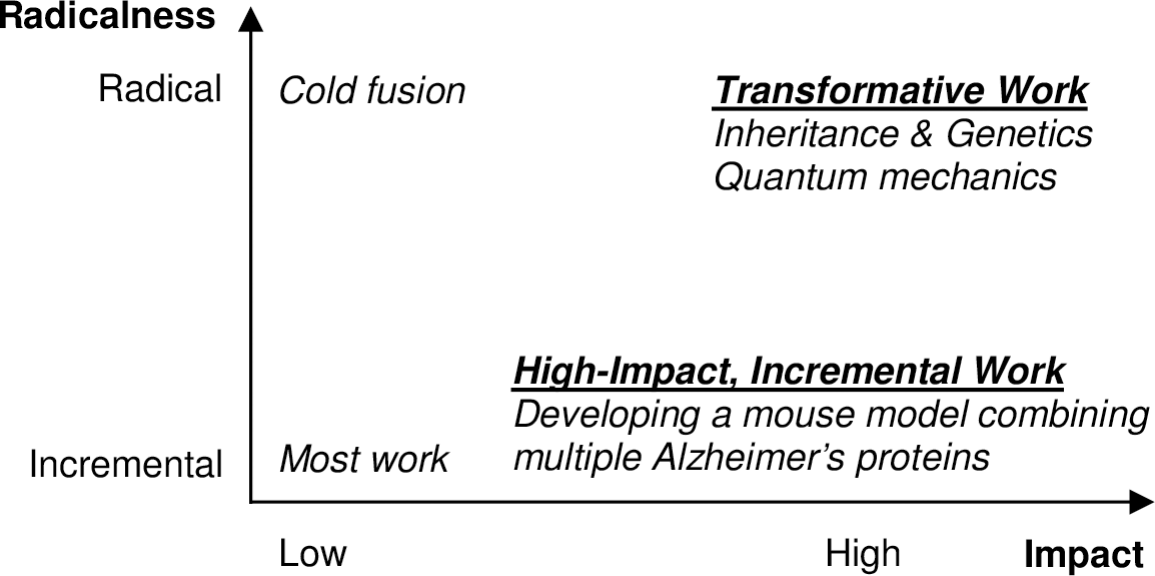
Classification of scientific work by radicalness and impact, with examples.

Consistent with the National Science Board’s description [1], scientific works vary continuously along two dimensions: 1) the extent to which they are radical (versus incremental) and 2) their impact, from low to high. These dimensions are illustrated in Fig 1. Most work in science is incremental, increasing knowledge and practices within an established paradigm or theoretical framework. As knowledge, products, and practices accumulate incrementally, moderate amounts of knowledge and practices become obsolete. High-impact incremental work naturally has a large impact on a field but lies within an existing paradigm. Consequently, high-impact, incremental work does not make obsolete a large amount of research (relative to its impact). Radical work differs from incremental work in that it represents a break from an existing paradigm. The highest-impact radical work is transformative and game-changing, fundamentally altering a discipline, making existing theories, paradigms, and knowledge obsolete, or at least less salient. It also generates new research opportunities, potentially across many fields. Of course, not all radical work is impactful. Low-impact radical work neither contributes to an established paradigm nor successfully replaces one. Our distinction between incremental and radical work parallels the distinction between normal and revolutionary science [13]. We hypothesize that this classification applies to non-scientific innovation and across research motivations (as in [14]).

Fig 1 provides examples that illustrate our classification, although we caution that a rigorous classification requires formal metrics such as those proposed in this paper.

Lower-left quadrant: Most scientific work is incremental and has a comparatively low impact. For example, in genetics and related fields, the discovery that two genes interact to produce a particular phenotype often is a publishable result. Dissecting the molecular mechanism controlling gene expression, however, is a more difficult and significant advance; this is the type of finding that is published in the top journals in molecular biology and genetics, such as *Cell*, and that has a higher impact.

Top-right quadrant: Quantum mechanics is a canonical example of transformative work in the 20th century, as it marked a shift from classical physics, changed physicists’ view of the world, and impacted other fields, such as chemistry. Examples of transformative research in biomedicine range from a series of breakthroughs in genetics and inheritance arising from Mendel’s genetic theory, to the discovery of the link between the DNA and inheritance, to the identification of the structure of DNA, which paved the way for the mapping of the human genome and launched the fields of genetics and molecular genetics. While these are among the greatest scientific transformations, we regard transformativeness as a continuous phenomenon, with more modestly transformative works transforming a subfield. In research on Alzheimer’s Disease, the first transgenic mouse model with the complex of pathologies found in humans with Alzheimer’s Disease (i.e. brain degeneration, memory deficits / learning impairment, and amyloid deposits) was transformative [15,16].

Lower-right quadrant: Again, in the case of Alzheimer’s Disease, after the first transgenic mouse model, there were a series of subsequent mouse models with other proteins and that incorporated multiple proteins in a single mouse model. Many of these developments were highly impactful, greatly expanding research opportunities, especially for translational research. At the same time, they were the culmination of ongoing efforts and did not radically alter a scientific paradigm, making them highly impactful incremental research, rather than transformative.

Top-left quadrant: Low-impact radical works are works that fail or lead to dead ends (e.g., cold fusion; see [12]) and radical works that impact a small area or make a small advance to a paradigm.

Scientific contributions can be classified as “conceptual” (e.g., discovery of the DNA structure) or “technical,” involving the development of methods or tools. Our classification of scientific works applies to both. Insofar as a technical contribution incrementally improves existing techniques and does not radically alter practices or overturn a theoretical framework, paradigm, or body of knowledge, it will be incremental. A new tool or method that renders existing tools or methods obsolete or whose application directly changes the theoretical paradigm in use is transformative. The invention of the tunneling microscope was transformative because it enabled new inquiries that ultimately resolved longstanding, fundamental questions and created new bodies of knowledge and even new fields [17]. Another example of a transformative scientific discovery of a technical nature is the discovery in 1998 of RNA interference (RNAi), a natural process by which cells silence the activity of specific genes. Prior to the discovery of RNAi, nearly the only method available to disable a gene in mammals was by creating knockout or transgenic animal models (such as the Alzheimer’s mouse models mentioned above), a very time-intensive and uncertain process. RNAi-based gene suppression is now the state-of-the-art method by which scientists can "knock down" specific genes in cells to learn about gene function [18].

As indicated, we draw on existing conceptualizations of HITS from NIH, NSF, and the NSB in order to identify aspects of research that are seen as making it transformative. We then develop metrics to measure each aspect that we identify. These metrics are calculated for each field-period pair. Here we use the term “aspect” to refer to some characteristic of research that is seen as making it transformative and “metric” to refer to an empirical measure that we develop to quantify the extent to which research in a given field- period pair is high in terms of each aspect of transformative research.

In recent years, the NIH has established programs that specifically target transformative research. The objective of NIH’s Roadmap Transformative Research Projects Program (R01) is to support “exceptionally innovative and/or unconventional research projects with the potential to create or overturn fundamental paradigms. These projects tend to be inherently risky and may not fare well in conventional NIH review… The primary emphasis of the Transformative Research Award is to support research on bold, paradigm-shifting but untested ideas” [19]. The Common Fund's NIH Director’s Transformative Research Award is intended to "support research on bold, paradigm-shifting but untested ideas” [19]. The NSF defines transformative research as involving “ideas, discoveries, or tools that radically change our understanding of an important existing scientific or engineering concept or educational practice or leads to the creation of a new paradigm or field of science, engineering, or education. Such research challenges current understanding or provides pathways to new frontiers [20]”. It describes transformative research as “revolutionizing entire disciplines; creating entirely new fields; or disrupting accepted theories and perspectives—in other words, those endeavors which have the potential to change the way we address challenges in science, engineering, and innovation [21].” Because potentially transformative research challenges the research agendas of experts on review panels, it may not receive a fair hearing. Also, as the NSB notes, transformative research frequently crosses disciplinary lines, adding to the challenge of evaluating the work. Nonetheless, it views transformative research as being “of critical importance in the fast-paced, science and technology-intensive world of the 21st Century [1]” and thus should be of paramount importance in determining how scarce funding is allocated.

These descriptions point to seven aspects of transformative work, many of which appear in multiple conceptualizations, and are often described using the same vocabulary. We view these aspects as *potentially* characterizing transformative work, with the *actual* features of transformative work being an empirical question that we seek to address in this work. The aspects, and how they map back to the conceptualizations, are outlined in Appendix A, Table A.1. We outline below the seven aspects of transformative research and eleven metrics we develop to measure the aspects. Section 4 in *Methods* provides a more detailed description of the 11 metrics that support the measurement of the seven aspects. Appendix D, Table D.1 provides formal definitions. Ultimately, the seven aspects will be grouped into those that measure impact and those that measure transformativeness (all metrics for the other six aspects) in the *Comparison of Metrics* in Section 5. All metrics for transformative work are computed at the level of field-period pairs as described in Section 3.

Before laying out the seven aspects of transformative work and the metrics we develop to measure them, we introduce a few conventions. We develop metrics based on the introduction and use of important new concepts in the literature, which we identify using 1, 2, and 3-word strings or “n-grams.” We refer to the introduction of these n-grams (the first year they are used in an article in the MEDLINE corpus) as concepts and occurrences in subsequent years as mentions, which we abbreviate as “Ment.” We also develop metrics using citations, which we abbreviate as “Cite.” We use “forward citations” to refer to the citations that a focal article receives in future works and denote these with “F;” we use “backward citations” to refer to the past works that a focal work cites and denote these with “B.” We use “T” to identify time windows. We denote metrics for Age using “Age” and metrics for dispersion with “Herf” to indicate Herfindahl indices, a common dispersion measure in economics. The seven aspects of transformative research and our metrics for them are:

1. **Radical-Generative**—Transformative research is viewed as critical because it generates radical new paradigms, theories, perspectives, and fields. We measure the generative aspect of transformative research using the birth of heavily used new n-grams, measured by a metric called *Concepts*, and the utilization of important new n-grams, called *BMentT*, where *T* indicates the number of years (0, 3, 5, 10, ∞) since the n-gram was first used in an article.
2. **Radical-Destructive**—In creating radical new paradigms, transformative research is seen to render large portions of existing knowledge obsolete (or at least less salient). The age of backward citations (the age of the works referenced in a focal article), captured by a metric called *BCiteAge*, indicates the extent to which current research draws on prior work. Backward citation ages have been shown to contract during scientific revolutions [22].
3. **Risky**—Because it represents a substantial departure from prior work, the existing conceptualizations view transformative work as risky. The risky nature is one reason why transformative work might not receive the support that it merits in funding reviews and why it is especially important to be able to identify and support it. One natural measure of risk is the variance in forward citations received by the articles published in a field-period pair, here called *FCiteVar*. In addition to the riskiness of research in a field-period pair, this measure reflects differences in the importance of work done in a field stemming from other sources.
4. **Multidisciplinary**—Transformative work is viewed as more likely to draw on knowledge from many fields. We use Herfindahl indices to measure the breadth of fields that are cited in articles and call this metric *BHerfCite*. In addition, we generate metrics for the breadth of important new n-grams that the articles in a field-period pair draw on. Specifically, we define *BHerfMentT*, where *T* indicates the number of years (0, 3, 5, 10, ∞) since the n-gram was first used in the MEDLINE corpus that we analyze here.
5. **Wide Impact**—Just as transformative work is viewed as more likely to draw on a wide range of knowledge, it is seen to be more likely to have a wide impact. We measure the breadth of impact using Herfindahl indices of the range of fields that cite articles using a metric we call *FHerfCite* and the range of fields that use the n-grams introduced by articles using a metric called *FHerfMent*.
6. **Growing Impact**—Because it is radical, the impact of transformative work is seen to take a while to accumulate. We measure the time path of utilization of transformative work using the mean time elapsed between when an article is published and the forward citations it receives. We note that the mean forward citation ages can be high for articles whose citations decline over time, so long as they decline relatively slowly. We call this metric *FCiteAge*.
7. **Impact**—In order for a radical work to be transformative, it must be impactful, so we view this aspect of transformative work as somewhat definitional. Put differently, works that are as radical as transformative work, but that do not have the same impact will not transform fields. We define the metric *FCiteMean* as the mean forward citation count; the percentiles of the distribution of forward citation counts we define as *FCiteN*, where N indicates the percentile of the citation distribution (25, 50, 75, 90, 95, 99, 99.9, 99.99).

## 3. Data acquisition and preparation

Two datasets are used to construct and exemplify the eleven metrics: 1) MEDLINE® 2014 baseline files distributed by the National Library of Medicine (NLM) containing 22,376,811 articles published between 1809 and 2014 [23] and 2) 15,085,762 articles from the Clarivate Analytics’ Science Citation Index Expanded^TM^ (SCIE) published between 1950 and May 20, 2014, the day our data were acquired. After taking the intersection of the two data sources, we are left with 13,737,835 articles published between 1950 and 2014. See Table 1 for details.

**Table 1:** Article counts.

Data Source	Articles	With Restrictions
MEDLINE 2014 Baseline *Published 1809–2014*	22,376,811	20,667,693*
SCIE	15,085,762	15,080,131**
*Published 1950-May 20*, *2014*		
Intersection		13,737,835
Published 1983–2012		10,778,696

*There are three restrictions on articles in the MEDLINE data: 1) the article must be the first version of an article, 2) the article must have “MEDLINE” status, and 3) the article must be tagged with at least one 4-digit MeSH term. For details on the version and status of MEDLINE articles see NLM, 2016. For details on 4-digit MeSH terms see the description below and Appendix C.

**There is one restriction on articles in the SCIE data: A small number of our SCIE records map to a PMID to which other SCIE records map. We retain the earliest SCIE ID that maps to each PMID, reducing our SCIE articles by 5,631 or .037% of our 15,085,762 SCIE records.

We are interested in generating two sets of metrics—one based on text analysis and another based on citations patterns. Since article abstracts are important for generating our text-based metrics, and MEDLINE’s coverage of abstracts is poor before 1980, we limit our sample to articles published in 1983 or later. Since citations take time to accumulate and our data ends in 2014, we limit our sample to articles published in 2012 or earlier. As seen in Table 1, restricting our sample to articles published between 1983 and 2012 leaves us with 10,778,696 articles with which to compute our metrics.

### Field identification

The 10,778,696 articles in our analysis sample are tagged with Medical Subject Headings (MeSH) by reviewers at the National Library of Medicine that describe the content of the articles. We assign articles to particular fields on the basis of these independently-assigned MeSH terms (the average article is tagged with 11.92 terms). There are 27,149 raw terms in the 2014 MeSH vocabulary and they vary widely in their descriptive detail. For instance, some articles are tagged with general terms such as "Body Regions" and some are tagged with more detailed terms such as "Peritoneal Stomata". In order to construct comparable fields, we aggregate all MeSH terms to a similar level of descriptive detail. This process—described in detail below and in Appendix C—leaves us with 6,159 aggregated MeSH terms.

To understand our aggregation method, first note that MeSH terms have a hierarchical structure. At the top of the hierarchy (1-digit terms) are 16 very general terms such as "Anatomy", "Organisms", and "Diseases". Beneath each of these categories, which we refer to as “1-digit MeSH terms”, is a group of more detailed “2-digit MeSH terms”. For instance, "Body Regions" is a 2-digit MeSH term beneath the 1-digit term "Anatomy". Beneath each 2-digit MeSH term is a group of even more detailed “3-digit MeSH terms”. This structure continues through 12 layers. To reduce the amount of variation in the breadth of fields, we aggregate all MeSH terms to the “4-digit level,” which we refer to as the “MESH4” level. Aggregation is complicated by the fact that some more detailed (lower level) MeSH terms are associated with more than one higher-level 4-digit MeSH term. In these cases, we distribute (prorate) the weight of each more detailed (lower-level) MeSH term evenly across all of the 4-digit MeSH terms that are above it.

Once we have finished this aggregation process, we are able to transform each article's raw MeSH terms, which vary dramatically in terms of degree of aggregation, into 4-digit MeSH terms, which are considerably more uniform in terms of degree of aggregation. We then characterize the fields to which an article belongs by prorating the article equally across its 4-digit MeSH terms. Thus, each article is fractionally assigned to one or more 4-digit MeSH fields. Appendix C, Fig C.2 and C.3 show the distribution of the number of MeSH4 terms per article by publication year.

### Field-period pairs

All metrics for high-impact and transformative science (HITS) are defined for field-period pairs, i.e., a combination of a specific 5-year period and a specific MeSH field. Since there are six consecutive 5-year periods (starting with 1983–1987 and ending with 2008–2012) and 6,159 MeSH fields, there are 36,945 *potential* field-period pairs. Some potential pairs are dropped because the field did not yet exist in the given period or because it did not contain any articles in our MEDLINE-SCIE matched sample, causing some metrics to be undefined. Overall, we are able to analyze 15,051 *actual* field-period pairs.

Table 2 illustrates the use (mentions) of highly used n-grams (*BMentAll*) and the mean of forward citations (*FCiteMean*) for five relatively highly ranked fields, which are detailed above and below. As noted, not all fields exist in all years. For instance, a MeSH code for the Human Genome Project was first introduced in 1989, so data are available only from the 1988–1992 period onward. Pluripotent Stem Cells and Nuclear Reprogramming come into use even later. It is noteworthy that *BMentAll* increases over time because the number of n-grams increases, while *FCiteMean* declines in the latest years because the length of time over which citations can accrue is shorter, a factor we control below.

**Table 2:** Exemplary depiction of field-period pairs.

	1983–1987	1988–1992	1993–1997	1998–2002	2003–2007	2008–2012
Field						
***BMentAll* (Number of Mentions of Top (.01%) Concepts in Titles and Abstracts)**						
DNA Methylation	0.00	0.01	10.13	92.28	276.07	564.49
Embryonic Stem Cells	0.36	2.77	3.90	3.16	450.15	2641.27
Human Genome Project		4.85	6.66	17.78	11.17	11.79
Nuclear Reprogramming				0.00	9.88	313.47
Pluripotent Stem Cells				7.71	185.06	1301.76
***FCiteMean (Mean Forward Citation Count)***						
DNA Methylation	32.60	4.00	57.64	73.94	46.11	18.72
Embryonic Stem Cells	34.62	37.47	36.30	25.27	32.45	19.45
Human Genome Project		7.03	18.37	26.63	14.21	10.43
Nuclear Reprogramming					96.28	34.52
Pluripotent Stem Cells			3.00	58.16	67.37	26.14

All six time periods are shown, but only five of the 6,159 fields and two of 11 metrics. Take the case of DNA Methylation: the numbers for the 2008–2012 period indicate that the (prorated) articles on DNA Methylation in this period used 564.49 top .01% concepts and were cited 18.72 times on average in the subsequent years.

## 4. Methods

Eleven metrics grouped by seven different aspects of impact and transformativeness were introduced in Section 2; they are defined and operationalized here.

We develop citation- and text-based metrics to identify the impact and transformativeness of the articles published in a given field-period pair. It is important to note that, when constructing a metric for a particular field-period, we typically use both articles that belong and do not belong to that field-period. For instance, consider an article published in 1990 in the field “Pluripotent Stem Cells (PSC)”. This article belongs to the “PSC-1988-1992” field-period. However, when we count forward citations to that article, we use citations occurring in articles belonging to *any* field published from 1990–2014. We can think of the field-period for which we are constructing the metric as the “target” field-period and the field-period from which we draw articles to construct the metric as the “measurement” field-period. In the example above, “PSC-1998-1992” is the “target” field-period and “any field-1990-2014” is the “measurement” field-period.

To compute the text-based metrics, we begin with the full MEDLINE 2014 baseline files containing 22,376,811 articles published between 1809 and 2014. We index all words, word-pairs, and word-triplets (generically referred to as “n-grams”) that appear in the title or abstract of a MEDLINE article. Next, we extensively process these n-grams by eliminating stop words, stemming and lemmatizing each word, and applying a variety of other operations. Note that the n-grams overlap with MeSH terms but, because they are extracted from titles and abstracts, they are not generally MeSH terms (and they vastly outnumber MeSH terms). See Appendix B for details.

After processing the MEDLINE corpus, we take the intersection of the MEDLINE and SCIE database, obtaining the 13,737,835 articles in Table 1. This set of articles contains 109,912,224 unique n-grams. Next, we use article publication dates to identify the first year each n-gram is mentioned, that is, appeared in an abstract or title of an article. We call this an n-gram’s “vintage year”. Further restricting our sample to the 10,778,696 articles published between 1983 and 2012, we obtain 95,393,331 n-grams with vintage years between 1982 and 2012. Next, we count the number of times an n-gram is mentioned subsequent to its vintage year. To focus on the most important n-grams, we identify the top 0.01 percent of n-grams from each vintage (including all tied n-grams in the case of ties at the threshold)—a total of 10,229 top n-grams (including 589 due to ties) with vintages between 1983 and 2012. We use these top n-grams to construct our text-based metrics.

Next, we verbally define each of the eleven metrics we have developed to capture impact and transformativeness. The full name of each metric and its variable name (in parentheses and italics) as well as formal definitions are given in Appendix D, Table D1. Summary statistics for all metrics and all field-period pairs are presented in Table 3. This table also provides details on the number of field-period pairs for which each metric can be computed and information on which measurement periods and fields are associated with each of the metrics.

**Table 3:** Summary statistics for all metrics for time periods 1983–1987, …, 2008–2012 and all MESH4 fields in MEDLINE.

Metric	Mean	S.D.	Metric Description, Measurement Period and Fields
**Citation-Based Metrics**			
FCiteMean	22.311	12.658	Mean citations received across articles indexed in both MEDLINE and SCIE in a field-period pair during all subsequent years (including later years in the target period) through 2014.
FCite25	3.489	2.654	Quantiles of the distribution of citations received across all articles indexed in both MEDLINE and SCIE in a field-period pair across during all subsequent years (including later years in the target period) through 2014.
FCite50	9.838	6.075	
FCite75	23.768	13.331	
FCite90	50.266	27.708	
FCite95	78.93	44.035	
FCite99	192.539	111.834	
FCite99.9	586.448	393.070	
FCite99.99	1626.674	1623.510	
FHerfCite	0.979	0.005	A Herfindahl index of the disciplinary diversity of the citations that the articles indexed in both MEDLINE and SCIE in a field-period pair received during all subsequent years (including later years in the target period) through 2014.
FCiteAge	5.229	2.555	The mean time to citation across all of the articles indexed in both MEDLINE and SCIE in a field-period pair.
BHerfCite	0.979	0.006	A Herfindahl index of the disciplinary diversity of the articles referenced by the articles indexed in both MEDLINE and SCIE in a field-period pair. Data on references cover all previous years (including earlier years in the target period).
BCiteAge	9.642	2.603	The mean of the mean age of the works referenced across all articles in a field-period pair. The mean reference age of an article is calculated over all references by the article to all articles that are published in all previous years (including earlier years in the target period) without limitations, which include all MEDLINE and non-MEDLINE indexed articles in the SCIE.
FCiteVar	4802.822	14513.130	The variance in citations received across all articles in a field-period pair. Citations to an article are the sum of citations received from all articles published in all subsequent years (including later years in the target period) through 2014 that are indexed in both MEDLINE and SCIE.
**Text-Based Metrics**			
Concepts	32.281	72.141	The number of top .01% n-grams introduced by a field-period pair. These are measured by identifying the year and field(s) in which each n-gram is born (i.e., the n-gram’s vintage year and field(s)). Only includes articles indexed in both MEDLINE and SCIE.
BMent0	0.003	0.006	The number of articles belonging to a field-period pair that mention a top .01% n-gram with in the first T (T=0, 1, 3, 5, 10, all) years since the n-gram was first used. Only includes articles indexed in both MEDLINE and SCIE.
BMent3	0.037	0.033	
BMent5	0.085	0.078	
BMent10	0.294	0.258	
BMentAll	1.028	0.930	
FHerfMentions	0.996	0.008	A Herfindahl index of the disciplinary diversity of the use of the n-grams introduced by the articles in a field-period pair. The metric is constructed from all mentions in all articles published across all years subsequent to the vintage year (including later years in the target period) through 2012. Only includes articles indexed in both MEDLINE and SCIE.
BHerfMent0	0.911	0.183	A Herfindahl index of the diversity of n-grams used by the articles in a field-period pair in the first T (T=0, 1, 3, 5, 10, all) years since the n-gram was first used. Only includes articles indexed in both MEDLINE and SCIE.
BHerfMent3	0.969	0.066	
BHerfMent5	0.976	0.053	
BHerfMent10	0.981	0.465	
BHerfMentAll	0.983	0.046	

Note: There are 15,051 field-period pairs. In all cases, articles are prorated across fields according to MeSH terms.

### Radical–generative

#### Top concept births (*Concepts*)

To measure the generation of important new ideas, we measure how many of the top 10,229 n-grams identified in the previous section are produced by a MeSH4 field in a particular period. To construct this metric, we first assign each n-gram to a period on the basis of its vintage. For instance, all n-grams with a vintage between 2003 and 2007 are assigned to the 2003–2007 period. Second, we assign each n-gram to MeSH4 fields. To do this, we identify all articles that use a particular n-gram in the first year it was introduced (its vintage year) and then identify the MeSH4 fields of these articles. We then prorate the n-gram equally across these fields. Finally, we sum the number of top n-grams assigned to each MeSH4 field-period pair. Concepts *are expected to be increasing with the radicalness of work*.

#### Top concept mentions (*BMentT*)

To measure the utilization of important new n-grams, we identify how many times one of the top 10,229 n-grams identified in the previous section are used within *T* (*T* = 0, 3, 5, 10, and all prior years) years of the n-grams’s vintage. To construct this metric, we first identify all articles that use a top n-gram from any vintage. Second, we assign each article to a period on the basis of its publication year. For instance, all articles published between 1993 and 1997 are assigned to the 1993–1997 period. Third, we assign each article to MeSH4 fields by equally prorating the article across the fields with which the article is tagged. Fourth, we count the number of top n-grams introduced within the last *T* years used by each article. Finally, we sum across all articles assigned to each MeSH4 field-period pair. BMent*T are expected to be increasing with the radicalness of work*.

### Radical–destructive

#### Backward Citation Age (*BCiteAge*)

This measure reflects the age of the works cited in articles. Radical changes in paradigms can be associated with reductions in backward citation ages. Intuitively, radical changes make older work less relevant, reducing citations to it [22]. The age of a backward citation is the difference between the publication year of the citing article and the publication year of the cited article (backward citation). For each citing article, a mean backward citation age is constructed by averaging the ages of its backward citations. *BCiteAge* for a target MESH4 field and 5-year period is the average of the average backward citation age across all articles published in the target field-period pair. BCiteAge *decreases with destructive radicalness*.

### Risky

#### Variance of forward citations (*FCiteVar*)

In economics, the variance of the returns to an investment or asset are used as a proxy for the investment or asset's risk. Here the riskiness of articles published in a field-period pair is measured by the variance in forward citations they receive. This metric uses all subsequent years (including later years in the target vintage) without limitations. For example, suppose a target field-period contains three articles, each assigned exclusively to the target field. Now consider one case in which each article receives 30 citations, and a second case in which two articles receive no citations and one receives 90 citations. The field-period forward citation mean is the same in both cases (it is 30) but in the first case *FCiteVar* is 0 $\left(=\frac{1}{3}(3\times {\left(30-30\right)}^{2})\right)$ and in the second case *FCiteVar* is 1800 $\left(=\frac{1}{3}(2\times {\left(0-30\right)}^{2}+{\left(90-30\right)}^{2})\right)$. FCiteVar *ranges between 0 and infinity and increases with riskiness*.

### Multidisciplinarity

#### Herfindahl of backward citations (*BHerfCite*)

*BHerfCite* for a target MeSH4 field and 5-year period is an index of field dispersion of the articles cited by the articles published in that target field and period. *BHerfCite* is computed by squaring the total number of backward citations from each field, summing over the squares, and subtracting the result from 1. For example, if the articles published in a target field-period cited 1500 articles, 500 in each of three fields, the field-period's *BHerfCite* would be .667 $\left(=1-\left({\frac{1}{3}}^{2}+{\frac{1}{3}}^{2}+{\frac{1}{3}}^{2}\right)\right)$. BHerfCite *ranges between 0 and 1 and increases with multidisciplinarity—the breadth of fields from which the article draws*.

#### Herfindahl Index of the Breadth of Existing Concepts Used (*BHerfMentT*)

To measure the breadth of ideas that a field is drawing on, we use a Herfindahl index of the dispersion of the top n-grams used by the articles published in that target field and vintage period. For a set of n-grams from a given field and vintage period, we square each n-gram's share of total mentions. We then sum over the squares and subtract them from 1. We do this separately by the number of years (*T* = 0, 3, 5, 10, and all prior years) since each n-gram was first used. BHerfMent*T ranges between 0 and 1 and increases with the breadth of ideas from which the field draws*.

### Wide impact

#### Herfindahl Index of Forward Citations (*FHerfCite*)

*FHerfCite* for a target MESH4 field and 5-year period is an index of field dispersion of the articles citing the articles published in that target field and vintage. *FHerfCite* is computed by squaring the share of forward citations from each field, summing over the squares, and subtracting the result from 1. For example, if the articles published in a target field-period were cited by 1500 articles, 500 from each of three fields, the field-period's *FHerfCite* would be .667 $\left(=1-\left({\frac{1}{3}}^{2}+{\frac{1}{3}}^{2}+{\frac{1}{3}}^{2}\right)\right)$. FHerfCite *ranges between 0 and 1 and increases with breadth of impact*.

#### Herfindahl Index of the Breadth of the Future Use of Concepts Introduced (*FHerfMent*)

To measure the breadth of use of the top n-grams generated in a field, we use a Herfindahl index of the dispersion across fields in the future use of the top n-grams introduced in a field-period pair. For a set of n-grams from a given field and vintage period, we take the share of mentions in subsequent periods across all fields, square each field’s share of total mentions. We then sum over the squares and subtract them from 1. FHerfMent *ranges between 0 and 1 and increases with breadth of use of the n-grams generated in a field*.

### Growing impact

#### Forward Citation Age (*FCiteAge*)

This measure captures the typical length of time between when works are published and citations to that work occur. The age of a forward citation to a cited article is the difference between the publication year of the citing article (forward citation) and the publication year of the cited article. One limitation of this metric is that forward citation ages can be high even if citations to a work decline, so long as the rate of decline is slow. For each cited article, a mean forward citation age is constructed by averaging the ages of its forward citations. *FCiteAge* for a target MeSH4 field and 5-year period is a weighted average of the article averages of forward citation age across all cited articles published in the target field-period. FCiteAge *increases with growth of impact*.

### Impact

#### Mean Forward Citation Count (*FCiteMean*)

*FCiteMean* for a target MESH4 field and 5-year period is the average forward citation counts across all articles (including those that receive no citations) published in the target field and period. FCiteMean *increases with impact*.

#### Forward Citation Percentile (*FCite*N)

This series of metrics captures the impact as measured by forward citation counts at various percentiles of the distribution of forward citations. Formally, we rank articles in a target field-period pair by their forward citation counts (including those that receive no citations). *FCite*N is the forward citation count below which *N* percent of articles in a target field-period pair are found. For example, *FCite75* is the forward citation count at which 75 percent of the articles in the target field and period have fewer forward citations. *FCiteN* is constructed for N = 25, 50, 75, 90, 95, 99, 99.9 and 99.99. FCite*N captures the impact of the most cited articles in a target field-period and increases with impact*.

As indicated, articles may be assigned to more than one MeSH category. In calculating each metric for each MeSH category, we weight articles by the share of the article falling into that MeSH category.

## 5. Results: Comparison of metrics

Our analysis proceeds in three steps. The first step is to collapse the many metrics that we have developed into indices for the seven aspects of transformative research. The second step is to aggregate the metrics for transformativeness into a single metric. The last step is to analyze the interrelations between the metrics.

### Generation of metrics for each aspect of transformative work

The eleven general metrics were analyzed and compared using a factor analysis to identify different aspects of transformativeness. Because the forward citation rates are the conventional measure of impact or influence, we perform a factor analysis on the impact metrics as a group.

When conducting the factor analysis, we first compute the natural logarithm of one plus all metrics and then take deviations from field and period means. We eliminate all variation across fields to account for the fact that some fields are larger than others. We also want to eliminate the common time trends to account for the fact that our metrics trend over time due to their construction (i.e. citation rates rise over time, but articles in the latest periods have less time to be cited). We do this by regressing the natural logarithm of one plus each variable on a set of dummy variables for 4-digit MESH field and a set of dummy variables for 5-year period. Formally, let *M_fp_* denote the value of a metric for field *f* in period *p*; $\overset{⃑}{F_{fp}}$ denote a vector of field dummy variables (or fixed effects) equal to one for field *f* and zero for the other fields; and $\overset{⃑}{P_{fp}}$ denote a vector of period dummy variables (fixed effects) equal to one for period *p* and zero otherwise. We estimate

$$ln(M_{fp}+1)={\overset{⃑}{F_{fp}}}^{\prime }\beta +{\overset{⃑}{P_{fp}}}^{\prime }\gamma +\epsilon_{fp}.$$

We analyze the residuals from this equation, *ϵ_fp_*. Taking the natural logarithm of the metrics reduces weight on variations in the right tail of the metrics, which tend to be highly right-skewed (adding one is a commonly used approach to address values of zeros). The period dummy variables address the end of our outcomes data in 2014, with the various vintages being different lengths of time from being truncated. Eliminating such cross-period variation when estimating factor loadings means that changes in the overall level of the metrics from period to period do not influence the factor loadings. We run the same comparison dropping the 2008–2012 period and found similar results. The field dummy variables address differences across fields in characteristics such as size. Thus, large fields are likely to generate more citations and the concepts they originate are likely to be more heavily mentioned. Eliminating cross-field variation before estimating the factor loadings means that the metrics are not influenced by overall differences across fields.

For all subsequent analyses, the dataset comprises all 15,051 field-period pairs, and observations are weighted by the number of articles in that field-period pair.

**Fig 2:**
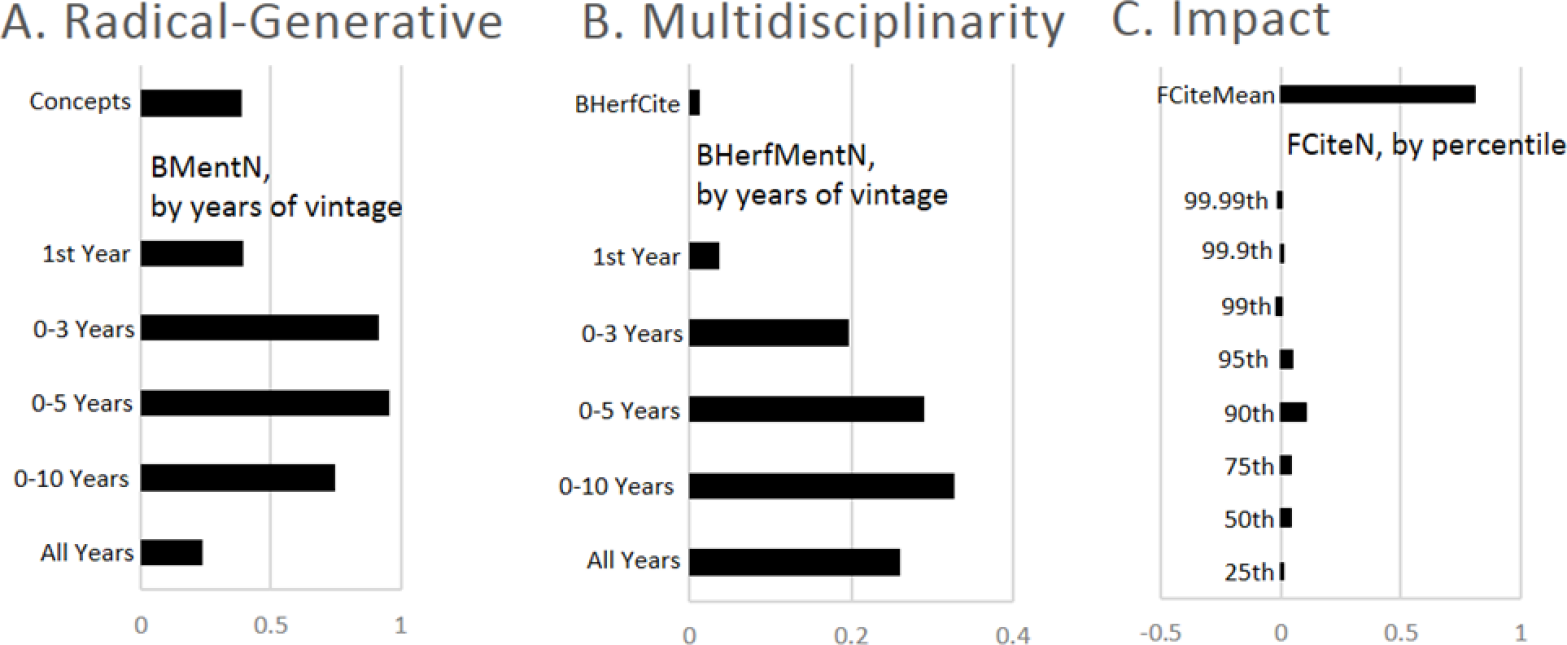
Factor loadings from a factor analysis results for three of the seven aspects.

Fig 2 reports results from the factor analysis for the three composite metrics–Radical Generative work, which combines *Concepts* and *BMentT*; Multidisciplinarity, which combines *BHerfCite* and *BHerfMentT*; and *Impact*, which combines *FCiteMean* and *FCiteN*. Note that the metrics for Radical Destructive work (*FCiteAge*), Risky work (*FCiteVar*), and Increasing Impact (*FCiteAge*) are all generated from a single metric, so that no factor analysis was performed. The metric for Breadth of Impact is based on only two metrics (*FHerfCite* and *FHerfMent*), and the factor analysis is not plotted. In all cases, the first factor accounts for the vast majority of the variation (74%-88%) and is the focal point here.

The figure indicates the extent to which the metrics for radical generative research, mutlidisciplinary research, and impact load on each of the sub-metrics (i.e. the factor loadings from the factor analysis). The share of variation explained by first factor is .741 for Radical-Generative work, .877 for Multidisciplinarity, and .830 for Impact.

Radical-Generative science (Fig 2A) loads positively on the birth of new n-grams (*Concepts*) and the mentions of important n-grams of various ages (*BMentT*), with the highest loading on n-grams that are 0–5 years old. Thus, n-grams that are older receive less weight than those that are 0–5 years old. N-grams that are younger receive more weight because they appear in multiple groups (i.e. a new n-gram appears in the 1^st^ year and years 0–3, 0–5, 0–10, and all). Thus, radical generative work draws on very young, very important concepts.

Multidisciplinary science (Fig 2B) loads positively on the dispersion of citations (*BHerfCite*) and the dispersion in the use of top n-grams (*BHerfMentT*), both measured using Herfindahl indices. The loading on n-grams that are 0–10 years old is the highest, but the weight on n-grams in their first year since origin is the highest because these n-grams are included in the other age categories, so the dispersion of the use of the newest n-grams is particularly related to multidisciplinarity.

Our metric for Impact (Fig 2C) is generated from the mean of forward citations (*FCiteMean*) and quantiles of the distribution of forward citations *FCiteN* (here constructed for N = 25, 50, 75, 90, 95, 99, 99.9 and 99.99), which tend to be closely related. The first factor of impact accounts for 83% of the variation. Mean Citations (*FCiteMean*) has the highest factor loading. Interestingly, the factor loadings on the quantiles of the forward citation distribution increase from the 25^th^ percentile of the citation distribution through the 90^th^ percentile and then decline, so that the lowest factor loadings are for the 99^th^ and the 99.99^th^ percentile of the citation distribution, which are slightly negative (the 99.9^th^ is positive but small, .00656). We show below that citations to the most highly cited papers are most likely to indicate transformative work, but impact in a field-period pair is more closely linked to the impact of highly impactful, but not works in the tail of the citation distribution.

Table 4 reports correlations between the various aspects of impact and transformativeness. The results show that many aspects of transformativeness are positively correlated, indicating some cohesion of these metrics of transformativeness. The Radical-Generative and Risky metrics are comparatively highly correlated (*ρ =* .*272*), suggesting that they are capturing inter-related phenomena. Both metrics are strongly positively correlated with Impact (*ρ =* .*384* and .*556*, respectively). Multidisciplinarity and Wide Impact research are also comparatively highly correlated (*ρ =* .*245*). It is intuitive and reassuring that work that draws on a wide range of work is itself drawn on by a wide range of work.

**Table 4:** Interrelations between the metrics for aspects of impact and transformativeness.

	Radical—Generative	Radical—Destructive	Risky	Multidis-ciplinary	Wide Impact	Growing Impact	Impact
**Radical–Generative**	1						
**Radical—Destructive**	0.1045	1					
**Risky**	0.2718	0.0501	1				
**Multidisciplinary**	-0.0752	-0.0963	0.0762	1			
**Wide Impact**	0.0676	-0.0167	0.0841	0.2472	1		
**Growing Impact**	-0.2948	-0.3529	-0.2322	-0.0344	-0.2428	1	
**Impact**	0.3835	0.0272	0.5558	0.1002	0.0343	-0.2000	1

Note: The table reports partial correlations between aspects of Impact and Transformativeness (the other six metrics) across field-period pairs after eliminating variation across field and time (that is, time and field fixed effects).

Other aspects of transformativeness appear to be only weakly related or unrelated. Radical-Generative is essentially uncorrelated with Wide Impact across disciplines (*ρ* = .068). Radical-Destructive is only weakly correlated with Radical-Generative (*ρ =* .*105*), suggesting strikingly that the generation of important new concepts in a field frequently occurs without rendering old science obsolete. Radical-Destructive is essentially uncorrelated with Risky (*ρ =* .*050*) and Wide Impact (*ρ = -*.*017*), but weakly negatively correlated with Multidisciplinarity (*ρ = -*.*096*). Interestingly, Multidisciplinarity is also not strongly correlated with Impact (*ρ =* .*100*) or Radical-Generative (*ρ = -*.*075*). These correlations contrast with the perspective that work that brings together differing scientific approaches or viewpoints generates more influential and radical scientific output.

The strongest correlation observed in Table 4 is between Impact and Risky (*ρ =* .*556*). We see a strong correlation between the variance in forward citation counts and citations at *all* quantiles of the citation distribution, including the 25^th^ percentile and the median, for example (not reported). This suggests the possibility of a trade-off between risk and return in scientific research.

As indicated, growth of impact over time, measured by the average time to citations, is negatively correlated with all the other metrics, which contrasts with the view that transformative work takes a long time to have an impact. We have broken forward citations to the work in each field-period pair into those arising in the first five years since publication and those arising six or more years since publication. Both metrics are positively related to each aspect of impact and transformativeness, but citations in the first five years are more strongly correlated with the other metrics for transformativeness (and impact) than are citations six or more years out: the correlation between transformativeness and citations in the first five years is .674, while that between transformativeness and citations six or more years after publication is only .247. Put differently, transformative work is heavily cited in the long run, but it is even more heavily cited in the short run. A limitation of the study is that we cannot measure impact over very long time periods; thus, we cannot rule out the possibility that the most transformative work grows in impact over much longer time horizons, e.g. over many decades.

### Aggregation of metrics into a single metric for transformativeness

**Fig 3:**
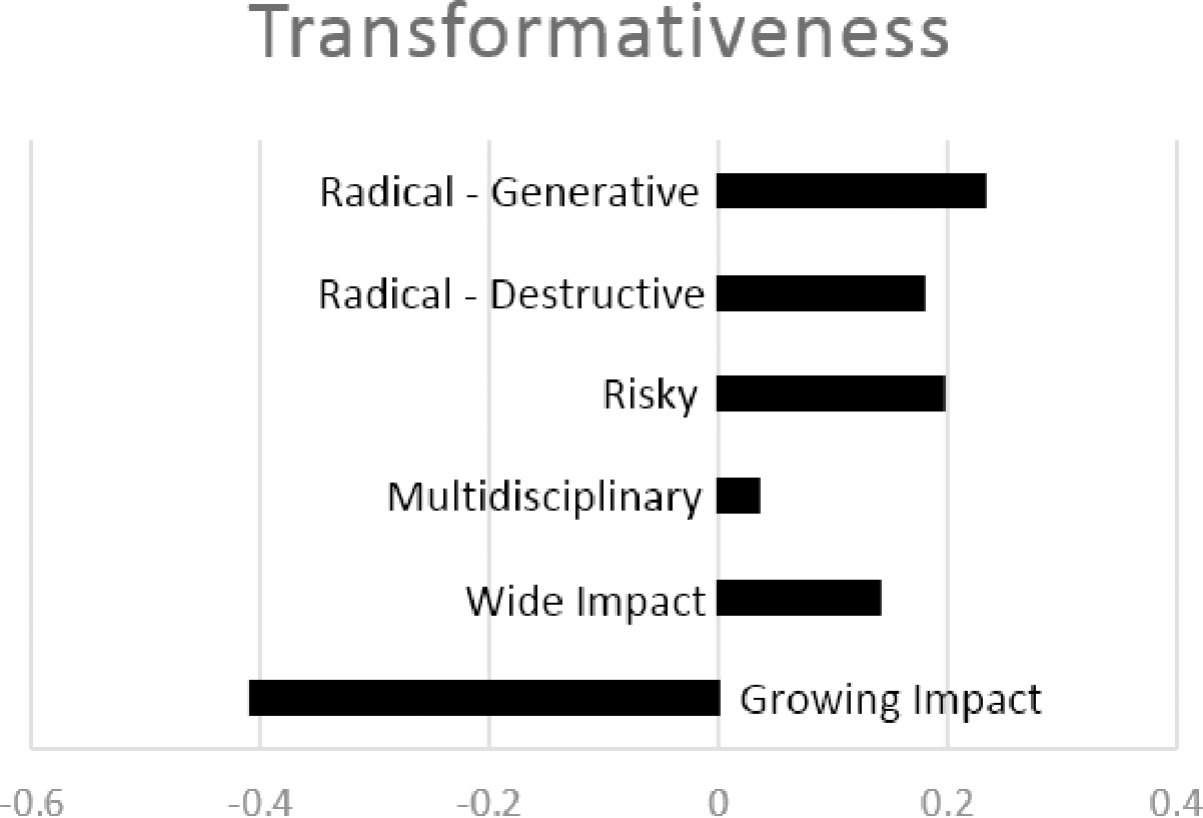
Results from a factor analysis of six aspects of HITS. The figure reports factor loadings on each aspect of transformative research from a factor analysis. The factor loadings indicate the extent to which the transformativeness metric loads on the (first) factor for each aspect of transformative research (excluding impact, which is treated separately).

Our next step is to aggregate the transformative metrics into a single metric. Fig 3 shows the first factor from a factor analysis of the six metrics of transformativeness (which accounts for 63% of the variation). Our metrics for Impact were excluded so that we can separately assess how impact and transformativeness are related. Transformativeness loads positively on all of the metrics except Growing Impact, suggesting that transformativeness represents a cohesive construct.

### Analysis of interrelationships between metrics

**Fig 4:**
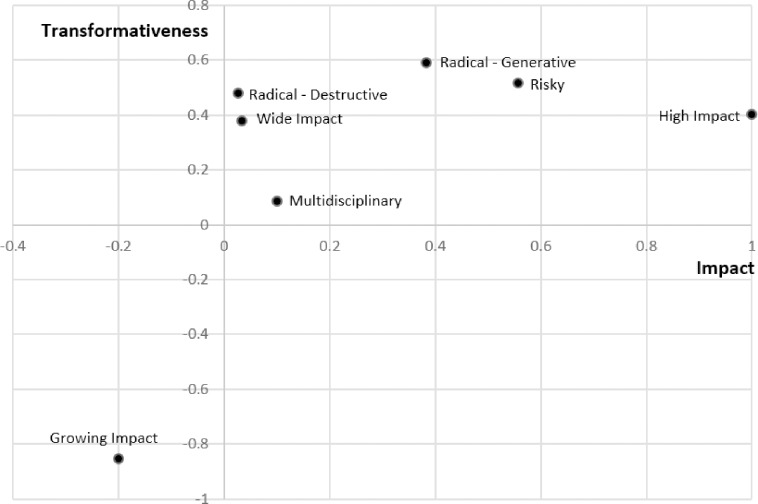
All seven aspects of HITS related to impact and transformativeness. The figure shows the partial correlations between the metrics for the aspects of transformative research and the overall metrics for transformativeness and impact across field-period pairs after eliminating variation across field and time (that is, time and field fixed effects).

Fig 4 relates the seven HITS metrics to our metrics for impact and transformativeness. Aside from Growing Impact, all the metrics of transformativeness are positively related to both impact and transformativeness. Looking across field-period pairs (and eliminating all time-invariant differences across fields and common changes over time), the metric for Impact has a partial correlation with itself of 1 (by construction) and a partial correlation with transformativeness of .402. Thus, while impact and transformativeness are positively related, they also seem to constitute distinct phenomena. The Risky metric is most strongly correlated with Impact. Radical-Destructive and Wide Impact are both strongly related to transformativeness but essentially unrelated to impact. Multidisciplinarity is weakly related to both impact and transformativeness. Lastly, Growing Impact is strongly negatively related to transformativeness and somewhat negatively related to impact. Interestingly, the correlation between impact and citations in the first five years is .881, falling slightly to .762 for citations six or more years after publication.

**Fig 5:**
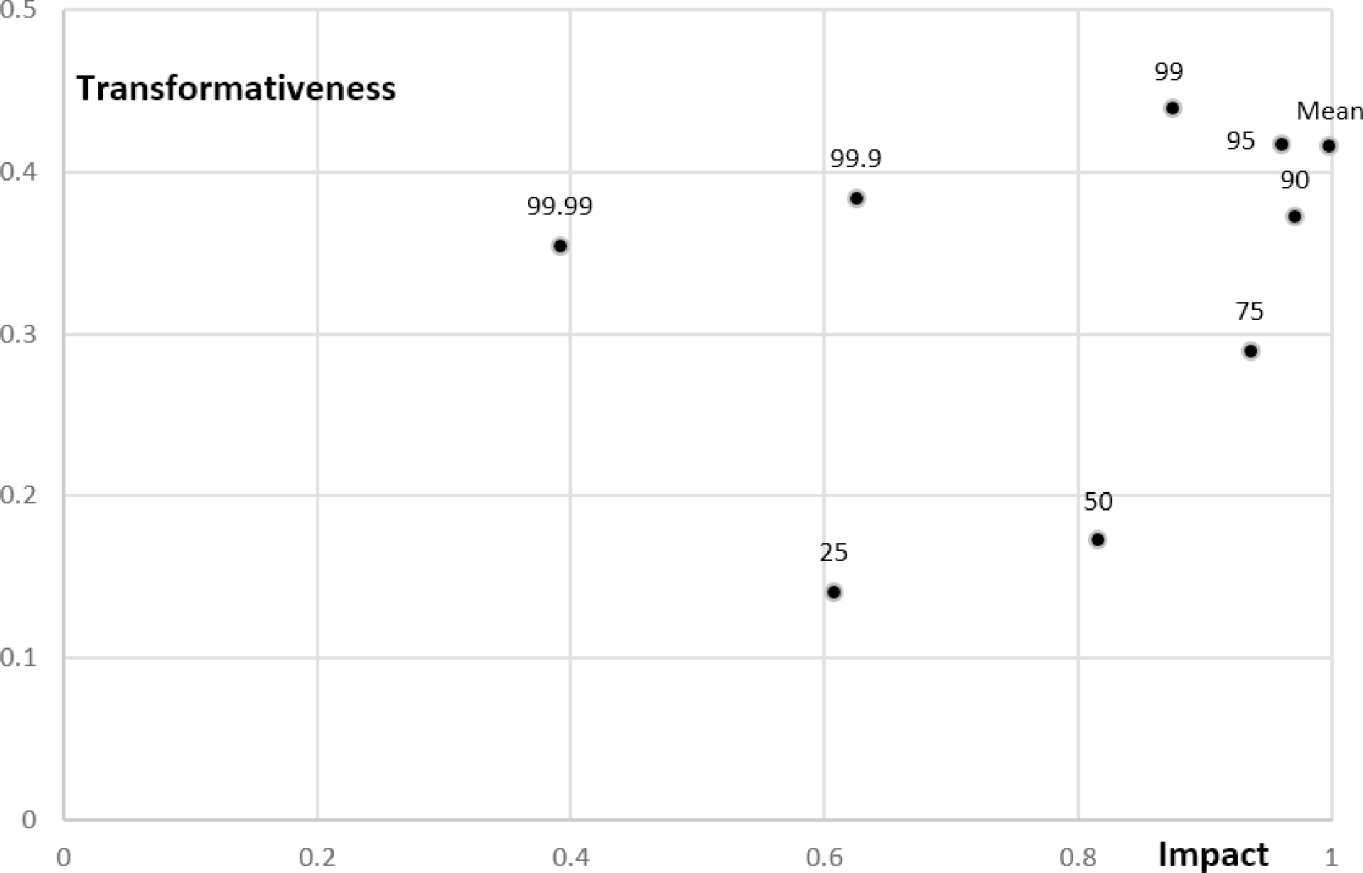
FCiteN related to impact and transformativeness. The figure shows the partial correlation between the individual metrics for impact and the overall metrics for transformativeness and impact across field-period pairs after eliminating variation across field and time (that is, time and field fixed effects).

Fig 5 shows how the various forward citation metrics (*FCiteMean* and *FCiteN*) relate to impact and transformativeness. As indicated in Fig 5, impact loads most heavily on mean citations and citations at the 90^th^ percentile with weight declining (or going negative) at the highest and lowest percentiles of the citation distribution. (The correlations between the impact measure and the highest percentiles of the citation distribution are positive even though the factor loadings are negative because all of the citation metrics are positively correlated.) It is intuitive that transformative works should be exceptionally highly cited. Indeed, the strength of the relationship between the percentiles of the citation distribution and transformativeness increases monotonically up to the 99^th^ percentile of the citation distribution (compared to the 90^th^ percentile for citations) and then declines moderately to the 99.99^th^ percentile. Strikingly, the 99.99^th^ quantile of the citation distribution is almost as strongly related to transformativeness as it is to impact. These results suggest that the most cited impactful works reflect a phenomenon distinct from other highly impactful works and that they are the most likely to be transformative.

**Fig 6:**
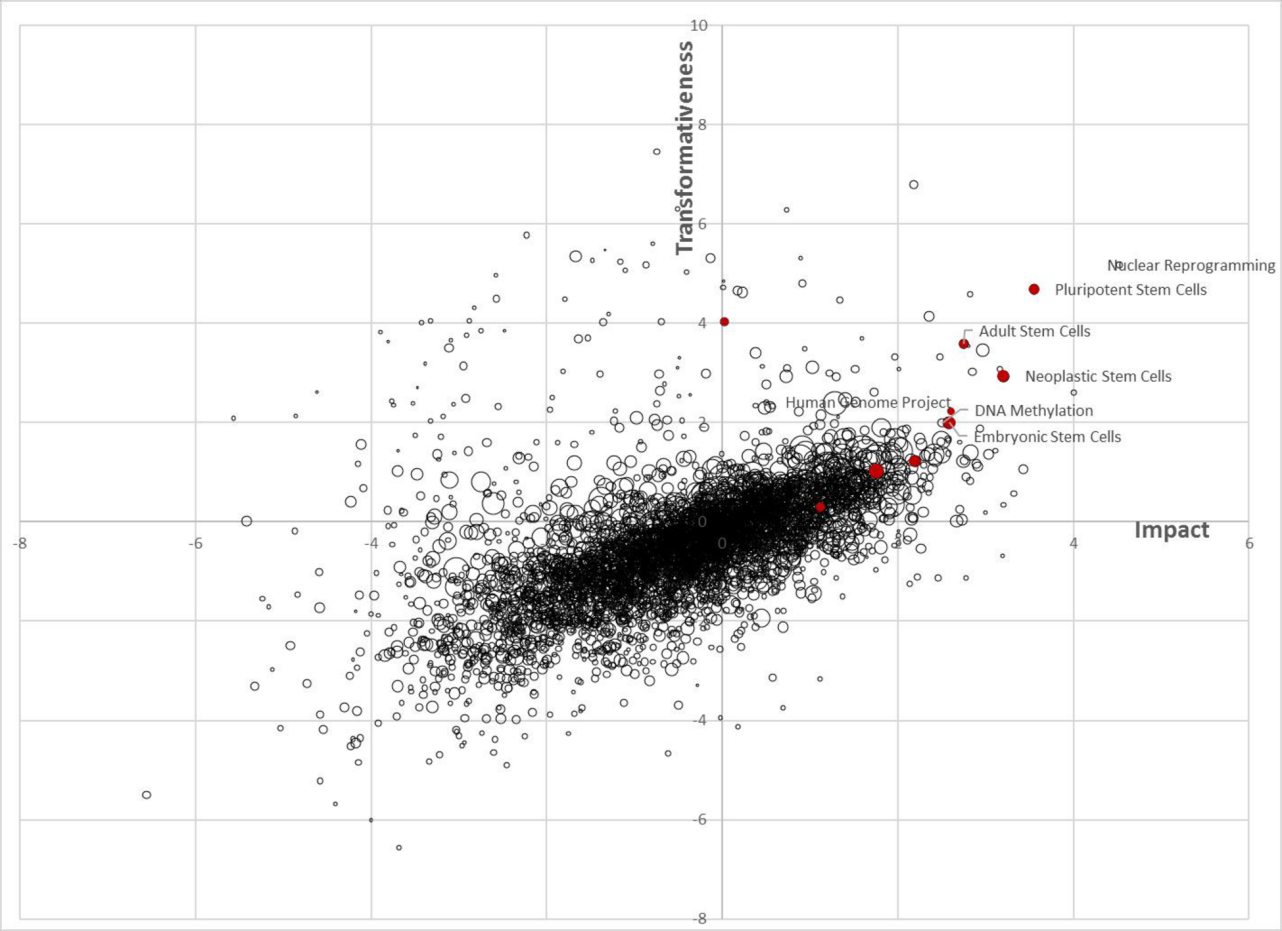
Ranking of fields in terms of impact and transformativeness across all periods (1982–2012). Field size determined by the number of (weighted) articles across all periods. Research on stem cells is shown in red.

To provide some summary of our analysis, we take the factor loadings from our factor analysis and use them to generate the impact and transformativeness metrics for each 4-digit MESH field, see Fig 6. In doing so, we average across all the periods from 1982–2012. While there are differences across fields, we do not eliminate field differences for this analysis. The figure shows a strong positive relationship between impact and transformativeness, but also differences. Here we highlight three examples. Research on stem cells is highlighted in red in Fig 6. It is widely viewed as potentially revolutionary because abnormal cell differentiation can be responsible for birth defects and cancer, because stem cells can assist in drug testing, and because of the potential for stem cells to generate new organs [24]. Epigenomics is critical for explaining differences in diseases and traits in the absence of differences in DNA sequences. Accordingly, work on cellular reprogramming and DNA methylation, both related to the accumulation and removal of epigenetic material that affects the functioning of genetic material without affecting the genome itself, ranks highly on transformativeness and impact [25]. This work has been recognized by *Science* as a Breakthrough of the Year repeatedly and has been supported by the NIH’s Common Fund, which seeks to support transformative research. The differences between impact and transformativeness can also be seen in the case of the Human Genome Project. As is well known, the Human Genome Project mapped the human genome and laid the foundation for the genomic revolution and advances in biotechnology. Strikingly, it ranks particularly highly on transformativeness (relative to impact).

Just as we preserve the cross-field variation in estimating the field rankings, we have rerun the factor analysis preserving the cross-field variation. The results are broadly similar to those reported above, with all the components of transformativeness entering in the same way as above. One clear difference is that the correlation between transformativeness and impact is higher when cross-field variation is preserved (*ρ =* .*696* versus .*402* when the cross-field variation is eliminated). This result is intuitive, in that it indicates that differences in transformativeness across fields are more strongly related to impact than are changes in transformativeness within fields over time. Put somewhat differently, fields that are transformative tend to be more impactful, while fields that are temporarily more transformative experience smaller increases in impact.

## 6. Discussion

The science policy community is increasingly focusing on transformative research, yet there are few metrics to identify transformative work even in retrospect and, ironically, the related concept of revolutionary science is falling out of favor. Drawing on existing conceptualizations of transformative research, this paper presented eleven metrics of transformative research. Specifically, transformative research is viewed as being radical, both generating important new ideas and destroying existing ideas; multidisciplinary and impacting a wide range of disciplines; risky; having a wide and growing impact over time; and being highly impactful.

Metrics for each of these aspects of high impact and transformative work were exemplarily applied to 15,051 fields of biomedical research over six five-year periods from 1983–1987 to 1988–2012. Many of the results from our analysis are intuitive, but some are unexpected. Our primary finding is that across fields and periods, impact and transformativeness are positively correlated but clearly represent distinct phenomena. This finding supports federal funding agencies’ separate emphasis on transformative research. The interrelations between specific metrics of transformativeness are often positively related but some are only weakly related or unrelated, suggesting that the seven aspects agencies conceive as integral to transformativeness are divisible and some appear to counter one another. Metrics of the use of wide-ranging ideas or multidisciplinarity are closely related to the breadth of impact. A notable exception we find is that the growth of citations is negatively related to transformativeness—while citations six or more years after publication are increasing in transformativeness, citations within the first five years increase even more. Whether this represents the limitations of the timespan of our data or a fundamental fact of transformative research, we leave to future research. In addition, we find that the displacement of old science coincides with the generation of radical new science only moderately, and that neither correlates strongly with multidisciplinarity, which is striking given the emphasis placed on multidisciplinary research. Interestingly, we find a strong positive association between impact and riskiness, which suggests the possibility of a trade-off between risk and return in scientific research.

Analysts have a number of choices when selecting metrics, with individual choices depending on data access and preferences, but also expertise and computational resources. All metrics for MEDLINE articles introduced in this paper are freely available for scholarly research subject to licensing restrictions (in the case of proprietary citation data). For those interested in generating our metrics over their own data or corpa, the titles and abstracts necessary to generate text-based metrics are openly available. The metrics of new concept births and mentions of concepts are relatively easy to compute, making it possible for anyone to compute metrics of radical generative work. The backward and forward Herfindahl indices of the breadth of mentions of new concepts have the same data requirements but are computationally more demanding. Thus, our text-based metrics of radical generative research, breadth of impact, and multidisciplinarity should be accessible to most practitioners. Generating citation metrics requires a different type of data access, e.g., to Clarivate Analytics’ Science Citation Index used here, or to one of the other citation databases. Calculating the mean citations to the works in a field-period pair, the quantiles of the citation distribution, and the variance of citations across the works in a field-period pair requires total citation counts to articles exclusively and is not computationally demanding. These provide good measures of impact and riskiness. As indicated, the extreme right tail of citations (e.g., the 99.99^th^ percentile of the citation distribution) is relatively strongly related to transformativeness and is not computationally burdensome either. The other citation metrics require data that go beyond raw forward citation counts, namely data on citing-cited article pairs. Backward citation ages and forward citation ages are both straightforward computationally, providing metrics of radical destructiveness and growth of impact. As with the text variables, the forward and backward citation Herfindahl indices are more computationally burdensome. Thus, while users must generate the metrics that suit their data access and computational environment, the tradeoffs they face when implementing our methods are obvious.

There are a number of limitations related to the data used and the metrics defined in this study. First, all of our analyses are limited in topical focus to articles published in MEDLINE and are limited temporally to the period 1983–2012. In terms of citation data from the Web of Science corpus, we used all backward citations of articles published in 1983–2012, yet we did not have access to forward citations beyond May 20, 2014. We used MEDLINE’s titles and abstracts for the text-based metrics exclusively. We used the MeSH hierarchy to measure the breadth of knowledge used in our target articles and the breadth of utilization of the ideas generated by our target articles and hence our definition of fields and our text-based metrics are restricted to the MeSH classification of the MEDLINE corpus. MEDLINE mostly covers biomedical research, a study of other research disciplines with different publication norms, researcher team sizes, and funding opportunities might provide different results. Given that our last period ends in 2012 and citation data ends in 2014, it is highly probable that the impact of some articles is still materializing. However, the results of our analysis seem robust, as omitting the 2008–2012 period does not change values dramatically.

There are a number of directions for future work. First, we look to validate our metrics in a variety of ways by soliciting feedback from subject matter experts in person and through surveys. We are also implementing an online interactive interface that users can visit to identify highly transformative research by selecting any of the eleven metrics and a time frame. A first version of the interactive interface can be found at http://cns.iu.edu/econ/hexmap.html (optimized to work with the Chrome web browser). Formal user studies have been run to examine what science map layout best supports memorization, search, and retrieval tasks [26]. User studies with domain experts will be run next to solicit feedback on the accuracy, representativeness, and usefulness of the HITS metrics. We are also interested in understanding how experts use the metrics and interactive visualizations to zoom in on subfields or make comparisons within and across fields, and over time. Ultimately, we would like to understand how experts would use the new metrics to improve their decision-making process.

Beyond validation, there are a number of other important avenues for future research. First, we are interested in identifying the factors—from the funding mechanisms, to the demographics of the researchers in fields, to the networks of researchers—that lead to the production of transformative science. Second, we seek to attach analogous metrics to individual articles, not just to entire research fields in a given period as we have done here. Such estimates would allow us to identify specific transformative works retrospectively. They would also allow us to identify features of research teams that have been associated with transformative work and that may facilitate its production in the future.

## Supporting information

S1 Appendix (DOCX)
