# Supplementary material for: High-impact and transformative science (HITS) metrics: Definition, exemplification, and comparison
Source: PLoS One. 2018 Jul 19;13(7):e0200597. doi: 10.1371/journal.pone.0200597 (PMC6053144; doi:10.1371/journal.pone.0200597)
Supplement: S1 Appendix — (DOCX) [file pone.0200597.s001.docx]

# Appendix

## A. Types and Metrics of Transformative Research

The below table identifies different types of transformative research and maps them to eleven metrics.

**Table A1.** **Types and metrics of transformative research**

| **National Science Board** | **NIH Roadmap** | **NSF** | **NIH Common Fund** |
| --- | --- | --- | --- |
| **Radical Generative** | | | |
| New paradigms or new scientific fields  generating new ones [paradigms] | Create … fundamental paradigms | Leads to the creation of a new paradigm … provides pathways to new frontiers. | Create … fundamental paradigms, bold, paradigm-shifting |
| **Metrics of generating important new concepts:**  Concepts (*Concepts*)—Introduction of heavily-used new n-grams  Mentions (*BMentT*)—Use of important new n-grams | | | |
| **Radical Destructive** | | | |
| Radically different approaches or interpretations;  overthrowing entrenched paradigms | Overturn fundamental paradigms | Radically change our understanding of an important existing scientific or engineering concept | Overturn fundamental paradigms |
| **Metrics of obsolescence of existing knowledge:**  Backward Citation Age (*BCiteAge*) (negatively related)—The age of prior knowledge drawn on | | | |
| **Risky** | | | |
|  | Inherently risky |  | Inherently risky |
| **Metrics of variation of new utilization of the knowledge generated:**  Variance of Forward Citations (*FCiteVar*)—Variation in utilization of knowledge produced | | | |
| **Multidisciplinary** | | | |
| Often can be multidisciplinary |  |  |  |
| **Metrics of drawing on a wide range of knowledge**  Backward Herfindahl of Citations (*BHerfCite*)—Breadth of articles used  Backward Herfindahl of Concepts (*BHerfMentT*)—Breadth of important n-grams used | | | |
| **Wide Impact** | | | |
|  | Impact a broad area of biomedical research |  |  |
| **Metrics of knowledge being used widely:**  Forward Herfindahl Citations (*FHerfCite*)—Herfindahl index of breadth of forward citations  Forward Herfindahl Mentions (*FHerfMent*)—Herfindahl index of breadth of future use of n-grams introduced | | | |
| **Growing Impact** | | | |
| Often fragile in their early stages |  |  |  |
| **Metrics of time path of utilization of knowledge:**  Forward Citation Age (*FCiteAge*)—Age of citations | | | |
| **Impact** | | | |
| Scientific understanding advances dramatically | Profoundly impact … biomedical research |  |  |
| **Metrics of citation impact:**  Forward citation counts (*FCiteMean, FCiteN*)—Number of citations | | | |

## B. Extracting and Processing Text

We first extract the title and abstract from each of the 22,376,811 records (articles) indexed in the 746 MEDLINE 2014 Baseline XML files. We then index all words, word pairs, and word triplets (1-, 2-, and 3-grams). We then process the n-grams contained in these titles and abstracts by performing the following operations:

1. Convert all text to lower case.
2. Eliminate 2- and 3-grams with words that cross the following characters: ,.?!;:)(\}\{][--.
3. Eliminate all remaining characters that are not alphanumeric.
4. Eliminate all n-grams that contain words appearing in the stopword list provided by the NLM at this address: https://mbr.nlm.nih.gov/Download/2009/WordCounts/wrd_stop
5. Eliminate all n-grams that contain the following character sequences: web, www, http, pubmed, MEDLINE.
6. Eliminate all n-grams that contain more than two adjacent numbers.
7. Eliminate all n-grams that have a length of less than three characters.
8. Keep all 1-grams with character length 3-29, 2-grams with character length 7-59, and 3-grams with character length 11-89.
9. Stem each word from each n-gram using the module *Lingua::Stem* from the Comprehensive Perl Archive Network (CPAN).
10. Index all the processed n-grams from each title and abstract into 746 tab-delimited text files corresponding to the 746 MEDLINE XML files.

As discussed in Section 3, only the 15,916,023 articles published in 1983-2012 are used to compute the text-based metrics. Here we focus on the 10,778,696 articles available in Clarivate Analytics’ the Science Citation Index Expanded^TM^ that match to MEDLINE.

## C. Aggregating MeSH Terms to Construct Fields

We use the Medical Subject Headings (MeSH) that tag most articles in MEDLINE to characterize the fields to which each article belongs. There are 27,149 raw terms in the 2014 MeSH vocabulary, and they vary widely in their descriptive detail. For instance, some articles are tagged with general terms such as “Body Regions” and some are tagged with more detailed terms such as “Peritoneal Stomata”. Thus, in order to construct comparable fields, we aggregate all MeSH terms to a similar level of descriptive detail.

To understand our aggregation method, first note that MeSH terms have a hierarchical structure. At the top of the hierarchy (first-level terms) are 16 very general terms such as “Anatomy”, “Organisms”, and “Diseases”. Each of these 16 first-level terms is identified by a unique capital letter. For instance, “Anatomy” is identified by the letter “A”, “Organisms” is identified by “B”, and so on. Beneath each of these first-level MeSH terms is a group of second-level MeSH terms. For instance, “Body Regions” is a second-level MeSH term beneath the top-level term “Anatomy”. Each second-level MeSH term is identified by the capital letter of the first-level MeSH term it is beneath and by two numbers. For instance, “Body Regions” is identified by “A01”. Beneath each second-level MeSH term is a group of third-level MeSH terms identified by the capital letter of the first-level term it is beneath, the two numbers of the second-level term it is beneath, and three subsequent numbers. For instance, “Anatomic Landmarks” is a third-level MeSH term under “Body Regions” and is identified as “A01.111”. This structure continues to depths of up to 12 levels.

Aggregating MeSH terms (that is, classifying lower level MeSH terms as a part of higher level MeSH terms) is not straightforward because some MeSH terms are beneath more than one higher level MeSH term and some articles can be tagged with multiple MeSH terms.

Consider the MeSH term “Asthma” This term has four separate identifiers: “C08.127.108”, “C08.381.495.108”, “C08.674.095”, and “C20.543.480.680.095”. Thus, “Asthma” falls under the first level MeSH term “Diseases” (identified by “C”). It also falls under the second-level terms “Respiratory Tract Diseases” (“C08”) and “Immune System Diseases” (“C20”). The problem arises because MEDLINE records contain only the MeSH terms themselves, not their identifiers. For instance, if a MEDLINE record is tagged with the MeSH term “Asthma”, we would not know whether it was the “Asthma” that is beneath “Respiratory Tract Diseases” or “Immune System Diseases”.

If we wanted to aggregate all MeSH terms to the second level, we would have to find a way to split “Asthma” between “Respiratory Tract Diseases” and “Immune System Diseases”. We opt for the straightforward method of assigning half to each higher level term. If we wanted to aggregate all MeSH terms to the fourth level, “Asthma” would fall under the fourth-level terms

- Lung Diseases, Obstructive [C08.381.495]
- Hypersensitivity, Immediate [C20.543.480]
- Asthma [C08.127.108]
- Asthma [C08.674.095]

In this case, we assign a quarter of the raw term “Asthma” to each of these four fourth-level terms. Thus, overall, 1/4 will be assigned to “Lung Diseases, Obstructive”, 1/4 to “Hypersensitivity, Immediate”, and 1/4+1/4=1/2 to “Asthma” itself.

Our last step is to apportion each article indexed in MEDLINE to the newly aggregated MeSH terms. Again, examples are the most illustrative. Suppose we come across an article that is tagged by “Asthma” and we want to aggregate to the fourth level. We know from above that 1/4 will be assigned to “Lung Diseases, Obstructive”, 1/4 to “Hypersensitivity, Immediate”, and 1/4+1/4=1/2 to “Asthma” itself.

However, suppose (as is usually the case) that this article is also tagged with other MeSH terms. Specifically, suppose that the article is also tagged with the terms “Neck” (identified by A01.598) and “Health Information Exchange” (identified by L01.700.253, L01.399.500.500, L01.313.500.500, and E05.318.308.940.968.625.500.500). Fig C.1 illustrates this example. By the process discussed above, 1/4 of “Health Information Exchange” will be assigned to each of the four fourth-level MeSH terms: “Health Information Exchange” itself (L01.700.253), “Health Information Management” (L01.399.500), “Medical Informatics” (L01.313.500), and “Data Collection” (E05.318.308). Since the lowest level of aggregation for “Neck” is the third level, we cannot assign it to a fourth-level term. We deal with this by simply eliminating “Neck”. It is too highly aggregated for our purposes.

We assume that each of the original remaining MeSH terms, “Asthma” and “Health Information Exchange”, receives equal weight in characterizing the article. Under this assumption, the article will be apportioned to each fourth-level MeSH term as follows:

- 1/2*1/4=1/8 to “Lung Diseases, Obstructive”
- 1/2*1/4=1/8 to “Hypersensitivity, Immediate”
- 1/2*1/4=1/8 to “Asthma”
- 1/2*1/4=1/8 to “Asthma”
- 1/2*1/4=1/8 to “Health Information Exchange”
- 1/2*1/4=1/8 to “Health Information Management”
- 1/2*1/4=1/8 to “Medical Informatics”
- 1/2*1/4=1/8 to “Data Collection”

Obviously, 1/8+1/8+1/8+1/8+1/8+1/8+1/8+1/8=1. Thus, the article that was originally tagged by the three MeSH terms “Asthma”, “Neck” and “Health Information Exchange” is now apportioned between eight different fourth-level MeSH terms. In general, we apportion each MEDLINE article across aggregated MeSH terms in two stages. First, we equally apportion the original MeSH terms across the higher-level MeSH terms of which they are a part (e.g. apportion “Asthma” equally across “Lung Diseases, Obstructive”, “Hypersensitivity, Immediate”, “Asthma”, and “Asthma”). Second, we weight this apportionment by the inverse of the number of original MeSH terms of the proper level that tag the article (e.g. our hypothetical article was tagged by three original MeSH terms, but only two at the proper level of aggregation, and so we weight by 1/2).


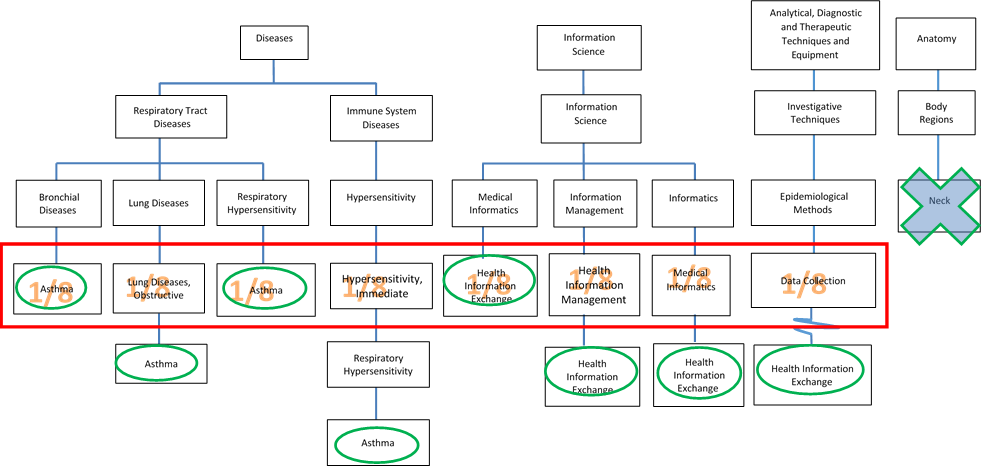


**Fig C.1. Process used to aggregate MeSH terms in order to construct fields.** Example shows MeSH terms “Asthma” and “Neck.”

Fig C.2 plots the distribution of 4-digit MeSH terms per article for the six different time periods. The number of MeSH terms assigned to articles increases over time.


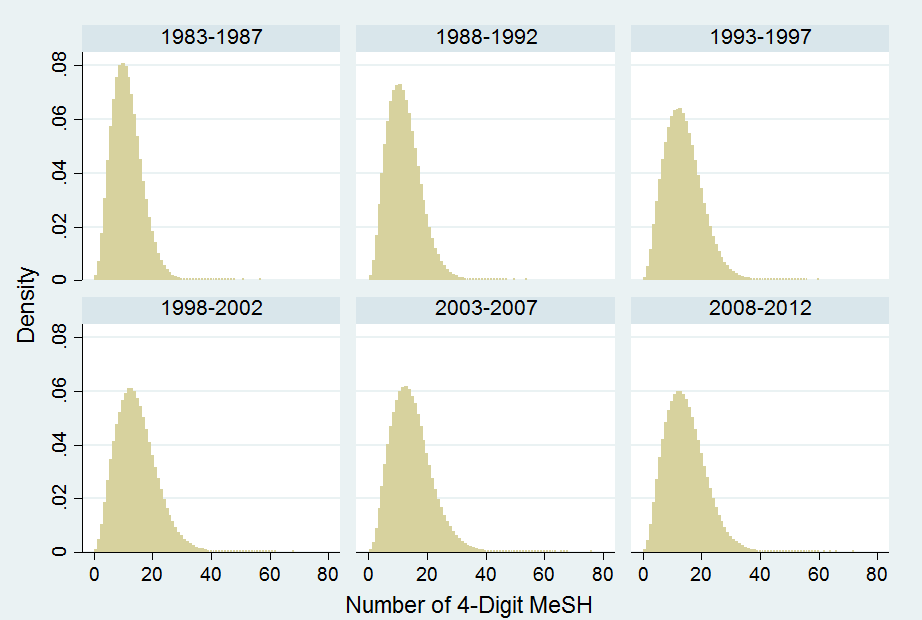


**Fig C.2.** **Distribution of the number of 4-digit MeSH terms per article.**

Fig C.3 shows the mean number of MeSH4 terms as well as selected quantiles (the 10th, 25^th^, 50^th^, 75^th^, 90^th^, and 99^th^ quantiles) of the distribution of the number of terms. The mean and median number of MeSH4 terms increases from roughly 10 per article in the 1970s to roughly 12 in 1974, after which the number of MeSH4 terms dips before gradually increasing to roughly 12 after 2000. In addition to the upward trend, there is an increase in dispersion of the number of MeSH4 terms, with no increase in the 10^th^ and 25^th^ quantiles in the most recent years.


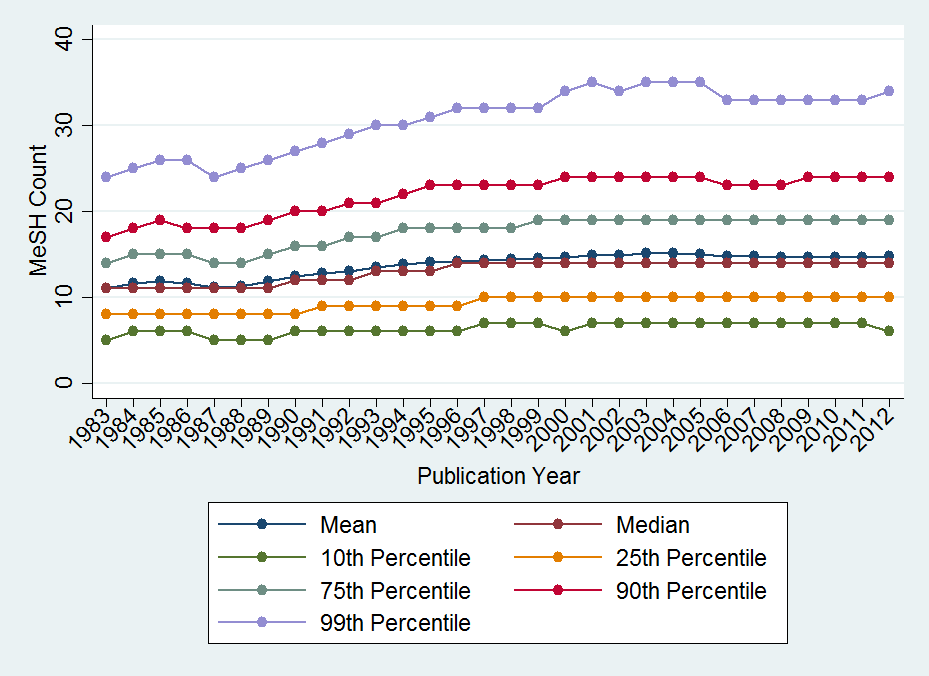


**Fig C.3. Quantiles of the distribution of the number of MeSH4 terms per article by publication year.**

## D. Formal Definition of Metrics

Common notation for the metric definitions include:

- $f$ denotes a field. When it is necessary to reference two fields simultaneously, $g$ can also denote a field.
- $p$ denotes a time period.
- $\mathbb{A}$ denotes a set of articles. We frequently use the set of articles published in field-period pair $fp$, which we denote $\mathbb{A}^{fp}$.
- $\mathbb{C}$ denotes the set of top n-grams (concepts). These are the top 0.01 percent of n-grams in terms of appearances in the titles and abstracts of MEDLINE from each vintage (including all tied n-grams in the case of ties at the threshold) — a total of 10,229 top n-grams (including 589 due to ties) with vintages between 1983 and 2012. We use these top n-grams to construct our text-based metrics.
- *N* denotes a count. For example, $N\left( \mathbb{A}^{fp} \right)$ denotes the number of articles in field-period pair *fp*
- We denote weights by $w$. For instance, $w\left( f|i \right)$ denotes the fraction of article $i$ that belongs to field $f$ and $w\left( i|fp \right)=\frac{w\left( f|i \right)}{\sum_{i\in\mathbb{A}^{fp}} w\left( f|i \right)}$ denotes article $i$’s share of all articles in field $f$ and period *p*.
- When identifying fields in which articles are cited, $\overline{f}$ indicates the field that is cited and $\underline{f}$ denotes the field that makes a citation.

**Table D.1. Formal definition of field-period metrics**

| 1. **Radical - Generative** | |
| --- | --- |
| Concepts | Let $c\in\mathbb{C}$ be a top n-gram. Let $i\in\mathbb{A}^{pc}$ be an article, published in period $p$, that uses $c$ in $c$’s vintage (birth) year. For instance, if $c$’s vintage is 2004, and $i\in\mathbb{A}^{pc}$, then $i$ is published in 2004 and uses $c$. Note that, in this example, $p$ corresponds to the period 2003-2007.  Since $w(f\vert i)$ is the fraction of article $i$ that belongs to field $f$, the total number of normalized articles that belong to field $f$ are published in period $p$, and use n-gram $c$ in $c$’s vintage (birth) year is $\sum_{i\in\mathbb{A}^{pc}} w\left( f \vert i \right)/{N(\mathbb{A}}^{pc})$.  Since $c$ is apportioned to a field in the same way as $i$, the total number of top n-grams that originate in field $f$ during period $p$ is defined as:  ${concepts}_{fp}=\sum_{c\mathbb{\in C}} \sum_{i\in\mathbb{A}^{pc}} \frac{w\left( f \vert i \right)}{N\left( \mathbb{A}^{pc} \right)}.$  Note that $w\left( f \vert i \right)$ is normalized by $N\left( \mathbb{A}^{pc} \right)$ to ensure that field-period pairs with many articles that use $c$ in its vintage year do not get counted as using more n-grams. |
| BMentT | Let $c\mathbb{\in C}$ be a top n-gram. Let $i\in\mathbb{A}^{pcT}$ be an article, published in period $p$, that uses $c$ within *T* years of $c$’s vintage (birth) year. For instance, if $c$’s vintage is 1999, $T=3$, and $i\in\mathbb{A}^{pc3}$, then $i$ is published any time between 1999-2001 and uses $c$. Note that, in this example, $p$ corresponds to the period 1998-2002.  Since $w(f\vert i)$ is the fraction of article $i$ that belongs to field $f$, the total number of (weighted) articles that belong to field $f$ are published in period $p$, and use n-gram $c$ within $t$ years of $c$’s vintage is $\sum_{i\in\mathbb{A}^{pcT}} w(f\vert i)$.  Since $c$ is apportioned to a field in the same way as $i$, the total number of top n-grams that are used within $t$ years of vintage and belong to field-period pair $fp$ is defined as:  ${BMent}_{fpT}=\sum_{c\mathbb{\in C}} \sum_{i\in\mathbb{A}^{pcT}} w\left( f \vert i \right).$ |
| 1. **Radical - Destructive** | |
| BCiteAge | Consider an article *j* that is cited by article *i.* The age of the citation to article *j* is  ${backward citation age}_{ij}=i^{'}s publication year-j^{'}s publication year$  and article *i*'s average backward citation age is  ${average backward citation age}_{i}=\frac{1}{\bar{N_{i}}}\sum_{j\vert i cites j} {backward citation age}_{ij}$  where $\bar{N_{i}}$ is the number of backward citations for paper *i*. Then the backward citation age among articles in field period pair *fp* is  $\mathrm{BCiteAge}_{fp}=\sum_{i\in\mathbb{A}^{fp}} w\left( i \vert fp \right)\times\left( {average backward citation age}_{i} \right).$ |
| 1. **Risky** | |
| FCiteVar | Let ${forward citation count}_{i}$ denote the count of forward citations to article *i* and $\mathrm{RawFCiteMean}_{fp}=\frac{1}{N\left( \mathbb{A}^{fp} \right)}\sum_{i\in\mathbb{A}^{fp}} {forward citation count}_{i}$, that is, the unfractionalized mean of forward citations in field period pair *fp.*  $\mathrm{FCiteVar}_{fp}=\frac{1}{N\left( \mathbb{A}^{fp} \right)}\sum_{i\in\mathbb{A}^{fp}} \left( raw {forward citation count}_{i}-RawFCiteMean_{fp} \right)^{2}.$ |
|  |  |
| 1. **Multidisciplinary** | |
| BHerfCite | Let $\mathbb{R}_{i\overline{f}}$ be the set of articles in field $\overline{f}$ that article *i* references. Then the weighted number of articles from field $\overline{f}$ that article *i* cites is $\sum_{k\in\mathbb{R}_{i\overline{f}}} w(\overline{f}\vert k)$.  Each article contributes to the backward citation count in field period pair *fp* according to its weight, $w\left( f\vert i \right)$ . Thus, the number of citations from all articles in field period pair *fp* to articles in cited field $\overline{f}$ is  $R_{fp}^{\overline{f}}=\sum_{i\in\mathbb{A}^{fp}} w\left( f\vert i \right) \left\{ \sum_{k\in\mathbb{R}_{i\overline{f}}} w\left( \overline{f} \vert k \right) \right\}.$  Summing $R_{fp}^{\overline{f}}$ over all fields gives the total number of backward citations from articles in field period pair *fp*:  $R_{fp}=\sum_{\overline{f}} R_{fp}^{\overline{f}}.$  Let $\omega_{fp}^{\overline{f}}=\frac{R_{fp}^{\overline{f}}}{R_{fp}}$ be the share of field period pair *fp*’s backward citations to field $\overline{f}$ . Then  $\mathrm{BHerfCite}_{fp}=1-\sum_{\overline{f}} \left( \omega_{fp}^{\overline{f}} \right)^{2}.$ |
| BHerfMentT | Let $c\mathbb{\in C}$ be a top n-gram. Let $i\in\mathbb{A}^{pcT}$ be an article, published in period $p$, that uses $c$ within $T$ years of $c$’s vintage (birth) year. For instance, if $c$’s vintage is 1989, $T=10$, and $i\in\mathbb{A}^{pc10}$, then $i$ is published any time between 1989-1998 and uses $c$. Note that, in this example, $p$ corresponds to the period 1988-1992.  Since $w(f\vert i)$ is the fraction of article $i$ that belongs to field $f$, the total number of articles that belong to field $f$, are published in period $p$, and use n-gram $c$ within $T$ years of $c$’s vintage is $n_{fpcT}=\sum_{i\in\mathbb{A}^{pcT}} w(f\vert i)$.  Since $c$ is apportioned to a field in the same way as $i$, the total number of n-grams that are used within $t$ years of vintage and belong to field-period pair $fp$ is given by $m_{fpT}=\sum_{c\mathbb{\in C}} n_{fpcT}$.  The fraction of n-gram $c$ uses within$T$ years of vintage that belongs to field-period pair $fp$ is then given by:  $\frac{n_{fpcT}}{m_{fpT}}=\frac{\sum_{i\in\mathbb{A}^{pcT}} w\left( f \vert i \right)}{\sum_{c\mathbb{\in C}} n_{fpcT}}=\frac{\sum_{i\in\mathbb{A}^{pcT}} w\left( f \vert i \right)}{\sum_{c\mathbb{\in C}} \sum_{i\in\mathbb{A}^{pcT}} w(f\vert i)}.$  The backward-looking Herfindahl index for fie $f$ in period $p$ using n-grams within $T$ years of vintage is defined as:  ${BHerfMent}_{fpT}=1-\sum_{c\mathbb{\in C}} \left\{ \frac{n_{fpcT}}{m_{fpT}} \right\}^{2}$ $=1-\sum_{c\mathbb{\in C}} \left\{ \frac{\sum_{i\in\mathbb{A}^{pcT}} w(f\vert i)}{\sum_{c\mathbb{\in C}} \sum_{i\in\mathbb{A}^{pcT}} w(f\vert i)} \right\}^{2}.$ |
| 1. **Wide Impact** | |
| FHerfCite | Let $\mathbb{Z}_{i\underline{f}}$ be the set of articles in field$\underline{f}$ that cite article *i*. Then the weighted number of articles from field $\underline{f}$ that cite article *i* is $\sum_{k\in\mathbb{Z}_{i\underline{f}}} w(\underline{f}\vert k).$  Each cited article contributes to the total forward citation count in field period pair *fp* according to its weight $w\left( f\vert i \right)$. Thus the number of citations from field $\underline{f}$ to all articles in field period pair *fp* is  $Z_{fp}^{\underline{f}}=\sum_{i\in\mathbb{A}^{fp}} w\left( f\vert i \right)\left\{ \sum_{k\in\mathbb{Z}_{i\underline{f}}} w\left( \underline{f} \vert k \right) \right\}.$  Summing $Z_{fp}^{\underline{f}}$ over all fields gives the total number of citations to articles in field period pair *fp*:  $Z_{fp}=\sum_{\underline{f}} Z_{fp}^{\underline{f}}.$  Let $s_{fp}^{\underline{f}}=\frac{Z_{fp}^{\underline{f}}}{Z_{fp}}$ be the share of field period pair *fp*’s forward citations in field $\underline{f}$. Then  $\mathrm{FHerfCite}_{fp}=1-\sum_{\underline{f}} \left( s_{fp}^{\underline{f}} \right)^{2}.$ |
| FHerfMent | Let $c\mathbb{\in C}$ be a top n-gram. Let $i\in\mathbb{A}^{pc}$ be an article, published in period $p$, that uses $c$ in $c$’s vintage (birth) year. Let $j\in\mathbb{A}_{\infty}^{c}$ be an article, published in *any* period, that uses $c$.  The fraction of $c$ originated in field $f$ is $w\left( f \vert c \right)=\sum_{i\in\mathbb{A}^{pc}} w\left( f \vert i \right)/{N(\mathbb{A}}^{pc})$. The total number of n-gram $c$ mentions by field $g$ (in any period) is $n_{cg}=\sum_{j\in\mathbb{A}_{\infty}^{c}} w(g\vert j)$.  The fraction of all weighted n-gram $c$ mentions by field $g$ is given by:  ${frac}_{fpcg}=\frac{w(f\vert c)*n_{cg}}{\sum_{g} \sum_{c\mathbb{\in C}} w(f\vert c)*n_{cg}}.$  The forward-looking Herfindahl index for field $f$ in period $p$ is defined as:  ${FHerfMent}_{fp}=1-\sum_{g} \sum_{c\mathbb{\in C}} \left\lceil{frac}_{fpcg} \right\rceil^{2}.$ |
| 1. **Growing Impact** | |
| FCiteAge | Consider an article *i* that is cited by article *j*. The age of the citation from article *j* is  ${forward citation age}_{ij}=j^{'}s publication year-i^{'}s publication year$  and article *i*'s average forward citation age is  ${average forward citation age}_{i}=\frac{1}{\underline{N_{i}}}\sum_{j\vert j cites i} {forward citation age}_{ij}$  where $\underline{N_{i}}$ is the number of forward citations for paper *i*.^2^  $\mathrm{FCiteAge}_{fp}=\sum_{i\in\mathbb{A}^{fp}} w\left( i\vert fp \right)\times\left( {average forward citation age}_{i} \right).$ |
| 1. **Impact** | |
| FCiteMean | $\mathrm{FCiteMean}_{fp}=\sum_{i\in\mathbb{A}^{fp}} w\left( i\vert fp \right)\times\left( {forward citation count}_{i} \right).$ |
| FCiteP | Order the $N\left( \mathbb{A}^{fp} \right)$articles in field period pair *fp* by forward citation count. Index the ordered articles by *j* = 1, 2, ..., $N\left( \mathbb{A}^{fp} \right),$ where *j* = 1 corresponds to the article with the highest forward citation count, *j* = 2 corresponds to the second highest citation count, and so on. Then compute  $\mathrm{FCiteP}_{fp}={forward citation count}_{z}$  $\mathrm{where}{forward citation count}_{z} is the forward citation count of the zth \mathrm{ordered}$  $article where$  $z=\arg\min_{j} \left\{ j, \mathrm{satisfies} \sum_{k=1}^{j} w\left( f\vert k \right)\geq(1-\frac{P}{100})\sum_{i\in\mathbb{A}^{fp}} w\left( f\vert i \right) \right\}$,  with *P* denoting the *P*^th^ percentile of the citation distribution. |
| Notes:  1. Top Concepts: A top concept is defined as one of the 10,128 most highly used 1-, 2-, or 3-grams that first appeared in a MEDLINE title or abstract between 1983 and 2012.   1. 2. FCiteAge: *forward citation age_ij_* is set to missing if either the value is negative or if *j*’s publication year is not valid. $\underline{N_{i}}$ is the number of forward citations to article *i* that have non-missing ages. | |
